# Olfactomedin-like 2 A and B (OLFML2A and OLFML2B) expression profile in primates (human and baboon)

**DOI:** 10.1186/s40659-016-0101-8

**Published:** 2016-11-08

**Authors:** Diana Cristina Pérez-Ibave, Rafael González-Alvarez, Margarita de La Luz Martinez-Fierro, Gabriel Ruiz-Ayma, Maricela Luna-Muñoz, Laura Elia Martínez-De-Villarreal, María De Lourdes Garza-Rodríguez, Diana Reséndez-Pérez, Jibran Mohamed-Noriega, Raquel Garza-Guajardo, Víctor Manuel Bautista-De-Lucío, Karim Mohamed-Noriega, Oralia Barboza-Quintana, Carlos Arámburo-De-La-Hoz, Hugo Alberto Barrera-Saldaña, Irám Pablo Rodríguez-Sánchez

**Affiliations:** 1 Servicio de Oncología, Universidad Autónoma de Nuevo León, Hospital Universitario “Dr. José Eleuterio González”, Monterrey, Nuevo León Mexico; 2 Facultad de Medicina, Universidad Autónoma de Guadalajara, Zapopan, Jalisco Mexico; 3 Unidad Académica de Medicina Humana y Ciencias de la Salud, Universidad Autónoma de Zacatecas, Carretera Zacatecas-Guadalajara Km.6. Ejido La Escondida, Zacatecas, Mexico; 4 Departamento de Ecología, Facultad de Ciencias Biológicas, Universidad Autónoma de Nuevo León, 66451 San Nicolás de los Garza, Nuevo León Mexico; 5 Departamento de Neurobiología Celular y Molecular, Instituto de Neurobiología, Universidad Nacional Autónoma de México, Querétaro, Querétaro Mexico; 6 Departamento de Genética, Universidad Autónoma de Nuevo León, Hospital Universitario “Dr. José Eleuterio González”, 64460 Monterrey, Nuevo León Mexico; 7 Departamento de Bioquímica y Medicina Molecular, Facultad de Medicina, Universidad Autónoma de Nuevo León, Monterrey, Nuevo León Mexico; 8 Departamento de Biología Celular y Genética, Facultad de Ciencias Biológicas, Universidad Autónoma de Nuevo León, Monterrey, Nuevo León Mexico; 9 Departamento de Oftalmología, Universidad Autónoma de Nuevo León Hospital Universitario “Dr. José Eleuterio González”, Monterrey, Nuevo León Mexico; 10 Servicio de Anatomía Patológica y Citopatología, Universidad Autónoma de Nuevo León, Hospital Universitario “Dr. José Eleuterio González”, Monterrey, Nuevo León Mexico; 11 Departamento de Bioquímica y Medicina Molecular, Instituto de Oftalmología. Fundación de Asistencia Privada Conde de Valenciana IAP, Mexico, Mexico

**Keywords:** Olfactomedin, Eye, Old world monkey, Baboon, OLFML2

## Abstract

**Background:**

The olfactomedin-like domain (OLFML) is present in at least four families of proteins, including OLFML2A and OLFML2B, which are expressed in adult rat retina cells. However, no expression of their orthologous has ever been reported in human and baboon.

**Objective:**

The aim of this study was to investigate the expression of OLFML2A and OLFML2B in ocular tissues of baboons (*Papio hamadryas*) and humans, as a key to elucidate OLFML function in eye physiology.

**Methods:**

OLFML2A and OLFML2B cDNA detection in ocular tissues of these species was performed by RT-PCR. The amplicons were cloned and sequenced, phylogenetically analyzed and their proteins products were confirmed by immunofluorescence assays.

**Results:**

OLFML2A and OLFML2B transcripts were found in human cornea, lens and retina and in baboon cornea, lens, iris and retina. The baboon OLFML2A and OLFML2B ORF sequences have 96% similarity with their human’s orthologous. OLFML2A and OLFML2B evolution fits the hypothesis of purifying selection. Phylogenetic analysis shows clear orthology in OLFML2A genes, while OLFML2B orthology is not clear.

**Conclusions:**

Expression of OLFML2A and OLFML2B in human and baboon ocular tissues, including their high similarity, make the baboon a powerful model to deduce the physiological and/or metabolic function of these proteins in the eye.

## Background

The olfactomedin (OLFM) family is a group of glycoproteins originally identified in bullfrogs (*Rana catesbeiana*) as the major component of the olfactory mucus layer, which surrounds the chemosensory dendrites of olfactory neurons [1]. Subsequently these proteins were found in the brain of species ranging from *Caenorhabditis elegans* to *Homo sapiens* [2]. OLFM shares a domain in the C-terminal of ~250 amino acids known as OLF [3]. Accordingly, in this domain, the OLF family was classified into seven subfamilies by phylogenetic analysis (as roman numerals from I to VII) [4]. Biological functions of proteins, which posses the OLF domain, remain for the most part elusive. A growing body of evidence indicates that these proteins may play very important roles in normal development and pathology [5]. For example, mutations in the OLF domain of myocilin were closely associated with primary open angle glaucoma [6]. Noelin-1 (OLFM-1) was found to play an important role in vertebrate neural crest development [7] and it is involved in frog neurogenesis (*Xenopus laevis*) [8]. Olfactomedin-likes (OLFML) are other members of the OLF family. Some of their members are the glycoproteins OLFML2A and OLFML2B, also known as photomedin-1 and photomedin-2, respectively, which were described in mice at 2005 [3]. OLFML2A and OLFML2B proteins are members of the subfamily IV [2]. The human OLFML2A and OLFML2B genes are located on chromosomes 9q33.3 and 1q23.3, respectively. These two genes are composed of at least 8 exons, and have a length of 37.3 and 40.7 kb, respectively [9].

In a mouse, OLFML2A and OLFML2B cDNAs have open reading frames (ORF) encoding 681 and 746 amino acid (aa) residues, respectively. Both proteins have a signal sequence at their N-terminal followed by two tandem CXCXCX_9_C motifs, a putative coiled-coil region, a serine/threonine-rich region, an OLF domain in their C-terminal, and two or three potential N-glycosylation sites. Both genes are expressed in the adult retina of mouse where they show mutually exclusive expression patterns. OLFML2A was predominantly detected in the photoreceptor layer, while OLFML2B is present in ganglion cells and inner nuclear layers, the inner segment of photoreceptor layer, and retinal-pigmented-epithelium [3]. Currently, the function of OLFML2 (A and B) proteins are still not clear and it is unknown if they are expressed in the retina of other mammals. Based on the above, the aims of this study were: (1) clone and sequence OLFML2A and OLFML2B cDNAs from the retina of baboons (*Papio hamadryas*) and humans; and (2) identify the cell layers where these proteins are expressed as a key to elucidate OLFML function in eye physiology.

## Methods

### Baboon’s biological specimens

Animal protocols were designed and developed according to the ethical guidelines of the Institutional Animal Care and Use Committee of the Texas Institute of Biomedical Research (TIBR). The baboon (*Papio hamadryas*) colony is preserved at the Southwest National Primate Research Center in San Antonio, Texas, USA. All the animals have the same diet and share similar environmental conditions. Baboons were gang-housed and fed ad libitum, a standard low-fat chow diet (Harlan Tecklad 15% monkey diet, 8715). The complete eyes of three adult female baboons (15, 16 and 18 years old) were collected. One eye was frozen in liquid nitrogen for RNA extraction and the other was included in formaldehyde at 4% for the immunofluorescence assays.

### Human’s biological specimens

Biopsies from human eyes were collected at the Department of Ophthalmology of the “Dr. José Eleuterio González” University Hospital of the Universidad Autónoma de Nuevo Leon, in Monterrey, Mexico. Specimens came from programmed eye surgery procedures where ocular tissues were removed by medical indication. All patients signed an informed consent according to the ethics committee guidelines of the institution. Biological samples were immediately immersed in RNA later solution (Ambion Inc., Austin, TX, USA) for RNA extraction and stored at −70 °C until their use. Paraffin-embedded biopsies, were used in immunofluorescence assays, these biopsies were provided by Pathology Department of the Instituto de Oftalmología Fundación de Asistencia Privada Conde de Valenciana IAP, at Mexico City. The characteristics of human eyes specimens are shown in Table 1.

**Table 1:** Characteristics of human eye tissues

Age (years)	Diagnostic/procedure	Eye tissues collected	Assay	Source
47	Enucleated eye for a traumatic penetrating injury	Retina, sclera and uvea	RT-PCR	1
82	Penetrating keratoplasty and cataract extraction for a cornea stroma scar and dislocated cataract	Lens, vitreous and corneal endothelium	RT-PCR	1
71	Enucleated eye for an orbit carcinoma, infective keratitis and neovascular glaucoma	Optic nerve	RT-PCR	1
7	Strabismus surgery (medical rectus recession)	Conjunctiva	RT-PCR	1
13	Strabismus surgery (inferior oblique muscle myectomy)	Inferior oblique muscle, conjunctiva and tendon	RT-PCR	1
2	Retinoblastoma	Conjunctiva, retina, choroid and sclera	IHF	2
35	Enucleated eye for a traumatic penetrating injury	Retina, uvea and sclera	IHF	2
63	Chorodial melanoma	Retina, uvea and sclera	IHF	2

*1* Department of Ophthalmology of the Hospital Universitario “Dr. Jose Eleuterio González of the UANL, *2* Department of Pathology of the Instituto de Oftalmología. Fundación de Asistencia Privada Conde de Valenciana IAP

### Reverse transcription and polymerase chain reaction

Each ocular piece from three adult baboons was dissected. The retina, cornea, lens, sclera, iris, choroid and optic nerve were separated. Total RNA was extracted from the eye tissues samples with Trizol reagent according to the manufacturer’s instructions (ThermoFisher Scientific Walthman, MA, USA). RNA was treated with RQ1 DNAse (Promega, Madison, WI, USA) for 15 min at 37 °C to remove traces of genomic DNA. For the assessment of RNA purity and integrity, we used standard methods of spectrophotometery and gel electrophoresis, respectively. Complementary DNA (cDNA) was synthesized using total RNA (1 µg), High Capacity cDNA Reverse Transcription kit (ThermoFisher Scientific) and oligo (dT) 12–18 primer (ThermoFisher Scientific) in 60 µL of total volume reaction. A primer set to amplify baboon OLFML2 A and B transcripts was designed using human sequences as templates; accesion numbers NM_182487 and AK316154, respectively. Such design was performed using an online tool [10], for OLFML2A the sense primer: 5′-CAGGCAGAGCGGGCGAAG-3′ and the anti sense primer: 5′-AATATTTGCGGACTGGGTCA-3′; while for OLFML2B, sense primer: 5′-AAGGGGCTGAGGACACTCTT-3′ and anti sense primer: 5′-GGAGGATGAGACCAGCACAT-3′. PCR was carried out using 100 ng of cDNA, 0.4 µM of each primer and GoTaq PCR master mix kit (Promega, Valencia, CA, USA). The amplification reaction was carried out in a thermal cycler Veriti 96-Well Thermal Cycler (ThermoFisher Scientific). The amplification used program was as follows: an initial denaturation step of 4 min at 94 °C, 40 cycles of 30 s each at 94 °C, 30 s at 60 °C, 90 s at 72 °C, and finally an elongation step of 6 min at 72 °C. The PCR products were visualized on 0.8% agarose gels stained with ethidium bromide and visualized under UV light.

### Molecular cloning and sequence analysis

The amplified products were cloned in the 3.5-kb XL-TOPO vector and transformed into electrocompetent *Escherichia coli* bacteria strain Top 10 according to the manufacturer’s specifications (Invitrogen, Carlsbad, CA, USA). Positive clones were sequenced using Big Dye Terminator Cycle Sequencing Kit v3.1 using specific oligos and/or M13 universal primers. The reactions were analyzed in the ABI PRISM 3100 Genetic Analyzer using the Sequencing Analysis Software v5.3 (Applied Biosystems, Foster city, CA, USA). The information obtained from the sequencing assays was subjected to a BLAST test to determine identity.

### Phylogenetic analysis

The sequences obtained from clones were aligned with the human orthologous reported gene (GenBank: NM_182487 and AK316154) using the CLUSTAL W program [11] followed by manual corrections in case of need. Protein sequences were derived by conceptual translation of the coding sequences. From amino acidic sequences, a phylogenetic tree was built with MEGA 6.06 software [12] using the maximum likelihood (ML), neighbor-joining (NJ) and UPGMA methods; then a bootstrap test was done with 1000 replicates [13]. Sequences used in this study are listed in Table 2.

**Table 2:** Primate OLFML2A and OLFML2B sequences from NCBI GenBank used in this study

Species	Accession no.			
	OLFML2A		OLFML2B	
	mRNA	Protein	mRNA	Protein
Apes				
Human (*Homo sapiens*)	NM_182487	NP_872293	AK316154	BAD38863
Chimpanzee (*Pan troglodytes*)	XM_520250	XP_520250	XM_513950	XP_513950
Pygmy chimpanzee (*Pan paniscus*)	XM_008960136	XP_008958384	XM_008978233	XP_008976481
Gorilla (*Gorilla gorilla*)	XM_004048596	XP_004048644	XM_004027786	XP_004027835
Orangutan (*Pongo abelii*)	XM_002820200	XP_002820246	XM_009241561	XP_009239836
Old world monkeys (OWM)				
Hamadryas baboon (*Papio hamadryas*)	KU587785	KU587785	KU587786	KU587786
Baboon (*Papio anubis*)	XM_003911245	XP_003911294	XM_009185676	XP_009183940
Rhesus monkey (*Macaca mulatta*)	XM_001082486	XP_001082486	XM_001118163	XP_001118163
Crab-eating macaque (*Macaca fascicularis*)	XM_005580795	EHH57082	XM_005541143	XP_005541200
Pig-tailed macaque (*Macaca nemestrina*)	XM_011711335	XP_011709637	XM_011770209	XP_011768509
Sooty mangabey (*Cercocebus atys*)	XM_012039189	XP_011894579	XM_012067471	XP_011922861
Green monkey (*Chlorocebus sabaeus*)	XM_008006272	XP_008004463	XM_007976370	XP_007974561
Angolan colobus (*Colobus angolensis palliates*)	XM_011931465	XP_011786855	XM_011952977	XP_011808367
Northern white-cheeked gibbon (*Nomascus leucogenys*)	XM_003264122	XP_003264170	XM_012511015	XP_012366469
Golden snub-nosed monkey (*Rhinopithecus roxellana*)	XM_010365852	XP_010364154	XM_010373616	XP_010371918
Drill (*Mandrillus leucophaeus*)	XM_011988676	XP_011844066	XM_011996103	XP_011851493
New world monkeys (NWM)				
Ma’s night monkey (*Aotus nancymaae*)	XM_012458157	XP_012313580	XM_012468868	XP_012324291
Marmoset (*Callithrix jacchus*)	XM_002743306	XP_002743352	XM_002760207	XP_002760253
Squirrel monkey (*Saimiri boliviensis boliviensis*)	XM_003940635	XP_003940684	XM_010331244	XP_010329546
Prosimians				
Gray mouse lemur (*Microcebus murinus*)	XM_012771871	XP_012627325	XM_012768612	XP_012624066
Coquerel’s sifaka (*Propithecus coquereli*)	XM_012639694	XP_012495148	XM_012639528	XP_012494982
Small-eared galago (*Otolemur garnettii*)	XM_003785297	XP_003785345	XM_003785760	XP_012658072
Philippine tarsier (*Tarsius syrichta*)	XM_008055582	XP_008053773	XM_008061396	XP_008059587

Seeking to identify the evolutionary forces that underlie the process of divergence in the OLFML2A and OLFML2B primate genes, we tested the hypothesis of positive or adaptive evolution (d_N_ > d_S_), purifying selection (d_N_ < d_S_), and neutrality (d_N_ = d_S_). For this purpose, first, we calculated the non-synonymous (causes an amino acid change) d_N_ and synonymous (does not cause an amino acid change) d_S_ distances respectively, by the the Li-Wu-Luo method (Kimura 2-parameters) [14] from OLFML2′ coding sequences from apes, OWM and NWM with their lemur counterpart. Second, we tested whether d_N_ is significantly greater, lower or equal, respectively, than d_S_ using a codon-based Z test of selection as implemented in MEGA 6.06 software [12]. Differences were considered statistically significant at a P < 0.05.

### Immunofluorescence assays

Ocular tissues were fixed in 4% paraformaldehyde (Sigma, St. Louis, MO, USA). These tissues were then dehydrated in a graded series of ethanol (50%, 30 min; 70%, 30 min; 95%, 30 min; 100%, 30 min) and cleared with xylene (Merck, Whitehouse Station, NJ, USA) for 30 min; tissues were then infiltrated with paraffin wax at 56 °C for 3 h. Serial transverse tissue sections (5 microns) were taken using a Leica RM-2135 microtome (Leica, Solms, Germany) and mounted into charged slides with 5% Gelatin (Bio-Rad, Hercules, CA, USA). The sections were cleared with citrosolv for 15 min and rehydrated with increasing serial ethanol concentrations (100, 100, 96, 96, 70, 70, and 50%) for five minutes each concentration and equilibrated in distillated water for 2 min. Exposure of the epitopes was performed by incubating the preparations with citrate buffer (10 mM sodium citrate, 0.05% Triton, pH 6). The sections were then blocked with 5% non-fat dry milk (Bio-Rad, Hercules, CA, USA); and treated with the reducing agent sodium borohydride in 0.01% in TBS 1X. The primary antibody incubation was overnight using a rabbit polyclonal anti-human OLFML2A antibody (Abcam ab75882, at a dilution 1:500), rabbit polyclonal anti-human OLFML2B antibody (Abcam ab75355, at a dilution 1:500). Double labeling was performed with both anti-OLFML2 antibodies and antibodies for neurons mouse monoclonal anti-human neuronal β-tubulin 3 beta chain (Covance MMS-453P, at a dilution 1:250) and astrocytes mouse monoclonal anti-human GFAP (anti-glial fibrillary acidic protein, Millipore MAB360, at a dilution 1:300). The antibodies were diluted in TTBS buffer with 1% skim milk. The primary antibody was omitted, for further negative controls. After primary antibody incubation, slides were washed with TTBS 1X buffer at room temperature for 10 min and repeating the process five times. Then the sections were incubated for 2 h in darkness at room temperature with secondary antibodies Cy3® goat anti-rabbit IgG (Life technologies, A10520, at a dilution 1:4000), Cy5® goat anti-rabbit IgG (Life technologies, A10523, at a dilution 1:4000), goat anti-mouse IgG FITC (Life technologies, 62-6511, at a dilution 1:250). The sections were washed five times with TTBS 1X for 5 min each time, subsequently placed with DAPI (Sigma, St. Louis, MO, USA, D-9542, at a final concentration of 0.1 µg/ml) for 20 min. The labeled sections were then washed three times with TTBS 1X for 5 min each time and mounted with Vectashield (Vector Laboratories, H-1000). Also, the labeled sections were examined using a LSM 780 confocal microscope (Carl Zeiss, Göttin-gen, Germany) and lasers at excitation wavelengths of 550, 650 and 495 nm, respectively. Digital images were brightness and contrast balanced.

## Results

### RT-PCR, molecular cloning and sequence analysis

**Fig. 1:**
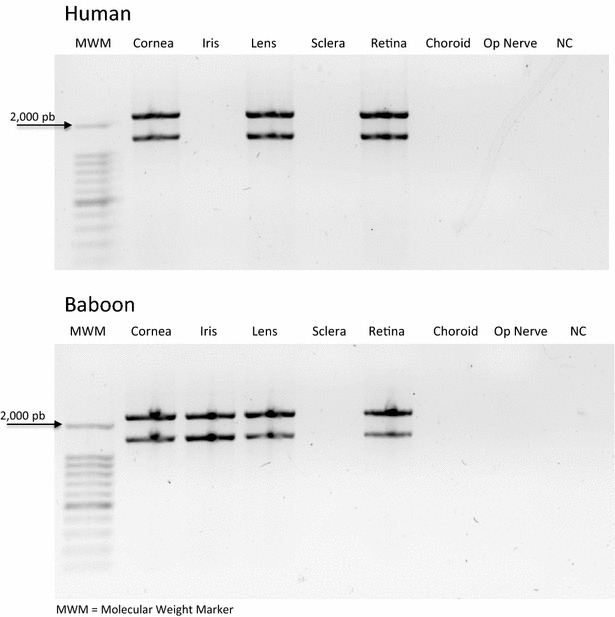
0.8% agarose gel. RT-PCR products were visualized and no other bans bans were detected as possible transcript variants. OLFML2A and OLFML2B were as expected 1947 and 2262 respectively

The OLFML2A and OLFML2B coding sequences were as expected (1947 and 2262 bp, respectively) and no other bands were detected as possible isoforms. In human samples, we detected amplification in retina, cornea and lens. While in baboon, amplification was detected in retina, cornea, lens and iris (Table 3; Fig. 1). The novel baboon OLFML2A and OLFML2B mRNA sequences were deposited in the GenBank database under the accession numbers KU587785 and KU587786, respectively. Such sequences have full CDS, which codifies for predicted proteins of 648 and 753 amino acids in length, respectively. The baboon OLFML2A and OLFML2B CDSs nucleotide sequences have 96% similarity with the human orthologous. In the amino acidic sequence, the similarity between baboon and humans is 98%.

**Table 3:** Tissue distribution of OLFML2A and OLFML2B mRNAs and proteins in baboon eye

Primate	mRNA	Tissue				
		Retina	Cornea	Lens	Sclera	Iris
Baboon	OLFML2A	√	√	√	X	√
	OLFML2B	√	√	√	X	√
Human	OLFML2A	√	√	√	X	X
	OLFML2B	√	√	√	X	X

Condition	Protein	Cell type or layer (of retina)					
		Ganglion cell layer (GCL)	Inner plexiform layer (IPL)	Inner nuclear layer (INL)	Outer nuclear layer (ONL)	Rods layer (RL)	Pigmented epithelium (PE)
Baboon	OLFML2A	√	√	√	√	√	√
	OLFML2B	√	√	√	√	√	X
Human	OLFML2A	√	X	√	√	X	X
	OLFML2B	√	X	√	√	X	X

**Fig. 2:**
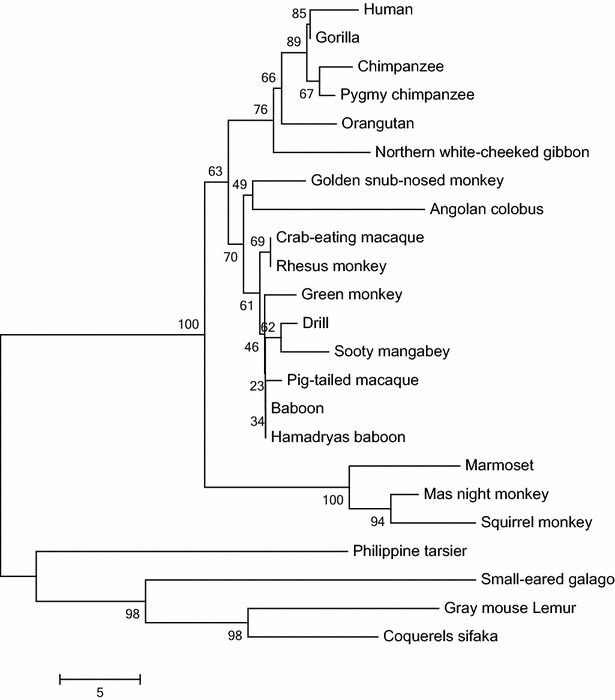
Phylogenetic tree of OLFML2A proteins from various primates. The tree was built using MEGA version 6.06 by the ML, NJ and UPGMA methods and further bootstrap analysis of 1000 replicas. The *numbers* in the *branches* indicate Bootstrap value and below the branches length. Clades are in linage specific manner, apes, OWM, NWM and lemur (out-group)

**Fig. 3:**
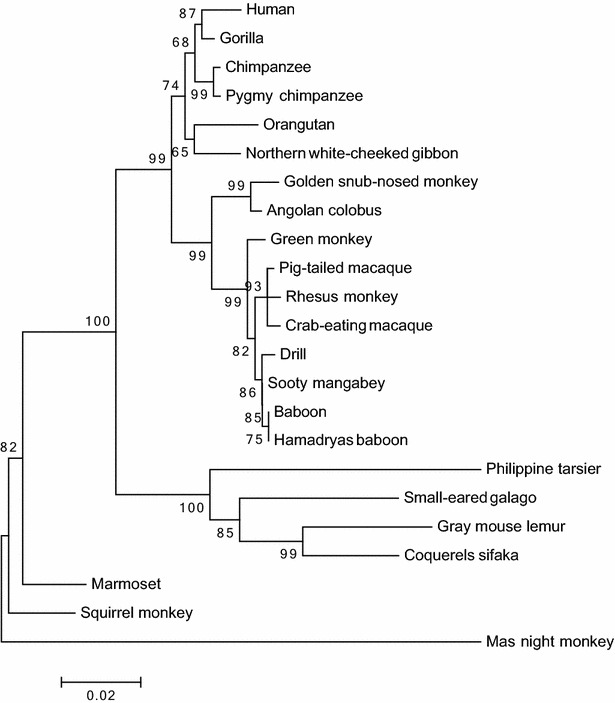
Phylogenetic tree of OLFML2B proteins from various primates. The tree was built using MEGA version 6.06 by the ML, NJ and UPGMA methods and further bootstrap analysis of 1000 replicas. *Numbers* in the *branches* indicate Bootstrap value and below the branches length. Clades are not in linage specific, NWM looks like out-group

Two phylogenetic trees were built, one for each protein. The OLFML2A tree (Fig. 2) shows four clades in a lineage-specific manner. These clades match in a lineage-specfic manner, they correspond to apes, OWM, NWM, and lemur (out-group). It confirms the orthology between primate OLFML2A genes. While the OLFML2B tree (Fig. 3) shows the same clades, but they are not in a lineage-specific manner. NWM act as out-grop, thus orthology is not clear. Bootstrap values are shown on the tree’s branches. Similar results were obtained using maximum likelihood (ML), neighbor-joining (NJ) and UPGMA phylogenetic methods. We confirmed that OLFML2A and OLFML2B evolution fit the hypothesis of purifying selection (d_N_ > d_S_, P < 0.05). See Table 4.

**Table 4:** Evolutionary forces that underlie the process of divergence in the OLFML2A and OLFML2B primate genes

Species	OLFML2A					OLFML2B				
	d_N_	d_S_	d_N_ = d_S_	P value		d_N_	d_S_	d_N_ = d_S_	P value	
				d_N_ > d_S_	d_N_ < d_S_				d_N_ > d_S_	d_N_ < d_S_
Apes										
Human	NA	NA	NA	NA	NA	NA	NA	NA	NA	NA
Chimpanzee	0.005	0.036	1.000	1.000	0.0001	0.009	0.031	1.000	1.000	0.0001
Pygmy chimpanzee	0.004	0.031	1.000	1.000	0.0001	0.009	0.031	1.000	1.000	0.0001
Gorilla	0.003	0.026	1.000	1.000	0.0001	0.007	0.031	1.000	1.000	0.0001
Orangutan	0.007	0.074	1.000	1.000	0.0001	0.015	0.048	1.000	1.000	0.0001
Old world monkeys (OWM)										
Hamadryas baboon	0.008	0.135	1.000	1.000	0.0001	0.021	0.071	1.000	1.000	0.0001
Baboon	0.008	0.144	1.000	1.000	0.0001	0.021	0.071	1.000	1.000	0.0001
Rhesus monkey	0.008	0.138	1.000	1.000	0.0001	0.023	0.073	1.000	1.000	0.0001
Crab-eating macaque	0.008	0.138	1.000	1.000	0.0001	0.024	0.074	1.000	1.000	0.0001
Pig-tailed macaque	0.009	0.144	1.000	1.000	0.0001	0.023	0.076	1.000	1.000	0.0001
Sooty mangabey	0.011	0.141	1.000	1.000	0.0001	0.019	0.066	1.000	1.000	0.0001
Green monkey	0.010	0.141	1.000	1.000	0.0001	0.020	0.075	1.000	1.000	0.0001
Angolan colobus	0.019	0.151	1.000	1.000	0.0001	0.021	0.079	1.000	1.000	0.0001
Northern white-cheeked gibbon	0.008	0.088	1.000	1.000	0.0001	0.013	0.046	1.000	1.000	0.0001
Golden snub-nosed monkey	0.011	0.135	1.000	1.000	0.0001	0.022	0.073	1.000	1.000	0.0001
Drill	0.010	0.135	1.000	1.000	0.0001	0.021	0.073	1.000	1.000	0.0001
New world monkeys (NWM)										
Ma’s night monkey	0.018	0.229	1.000	1.000	0.0001	0.128	0.241	1.000	1.000	0.0001
Marmoset	0.022	0.247	1.000	1.000	0.0001	0.034	0.145	1.000	1.000	0.0001
Squirrel monkey	0.022	0.221	1.000	1.000	0.0001	0.035	0.137	1.000	1.000	0.0001
Prosimians										
Gray mouse lemur	0.050	0.280	1.000	1.000	0.0001	0.060	0.327	1.000	1.000	0.0001
Coquerel’s sifaka	0.044	0.329	1.000	1.000	0.0001	0.054	0.232	1.000	1.000	0.0001
Small-eared galago	0.050	0.402	1.000	1.000	0.0001	0.057	0.297	1.000	1.000	0.0001
Philippine tarsier	0.045	0.392	1.000	1.000	0.0001	0.066	0.242	1.000	1.000	0.0001

### Localization of OLFML2A and OLFML2B proteins in the eye of baboons and humans by immunofluorescence assays

**Fig. 4:**
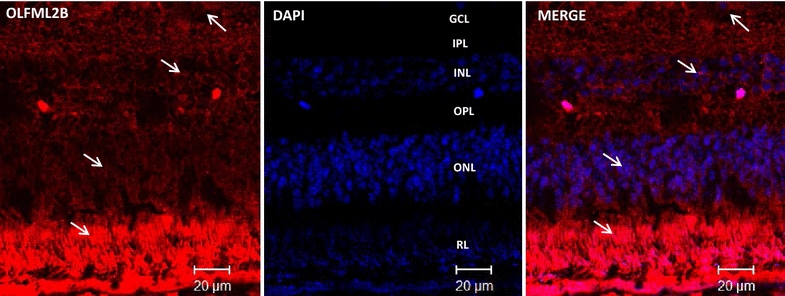
OLFML2A immunodetection in the retina of adult baboons. Confocal images of double stained retina sections to identify cells expressing OLFML2A (*red* 1st Ab: rabbit polyclonal anti-human OLFML2A 1:500; 2nd Ab: goat anti-rabbit IgG-Cy3® 1:4000), β-Tubulin (1st Ab: mouse monoclonal anti-mammal Tubulin 3 beta chain 1:250, 2nd Ab: goat anti mouse IgG FITC 1:250) and Glial Fibrillary Acid Protein (1st Ab: mouse monoclonal anti-GFAP 1:300, 2nd Ab: goat anti mouse IgG FITC 1:250) in baboon retina. Cells nuclei were labeled with DAPI (*blue*). *GCL* ganglion cell layer, *IPL* inner plexiform layer, *INL* inner nuclear layer, *ONL* outer nuclear layer, *RL* rod layer, *PE* pigmented epithelium

**Fig. 5:**
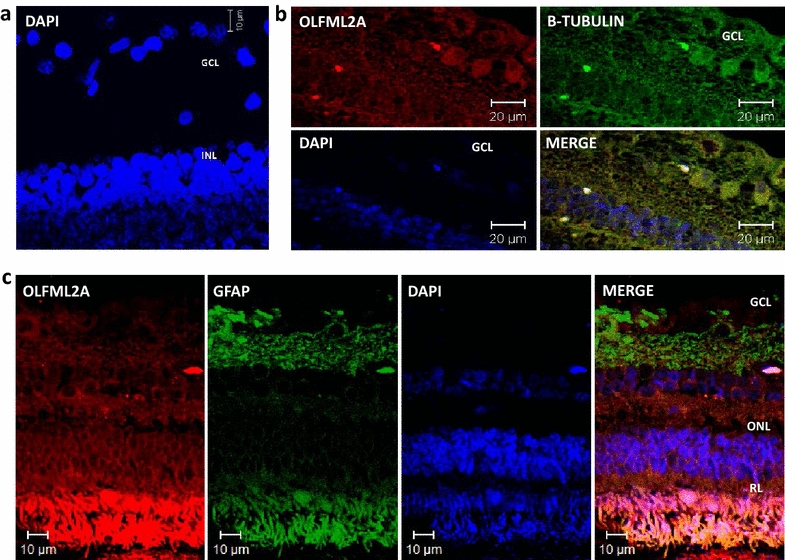
OLFML2A and OLFML2B double stained immunoreactivity in baboon retina.** a** Negative Control of baboon retina sections wherein the incubation with the 1st Ab is omitted and incubated with only 2nd Ab: goat anti-rabbit IgG-Cy3® 1:4000. **b** Confocal images of double stained retina sections to identify cells expressing OLFML2A (*red*, 1st Ab: rabbit polyclonal anti-human OLFML2A 1:500; 2nd Ab: goat anti-rabbit IgG-Cy3® 1:4000) and β-Tubulin (*green*, 1st Ab: mouse monoclonal anti-mammal Tubulin 3 beta chain 1:250) in baboon retina. **c** Confocal images of double stained retina sections to identify cells expressing OLFML2A (*red*, 1st Ab: rabbit polyclonal anti-human OLFML2A 1:500; 2nd Ab: goat anti-rabbit IgG-Cy3® 1:4000) and Glial Fibrillary Acid Protein (*green*, 1st Ab: mouse monoclonal anti-GFAP 1:300, 2nd Ab: goat anti mouse IgG FITC 1:250) in baboon retina. Cells nuclei were labeled with DAPI (*blue*).* GCL* ganglion cell layer,* INL* inner nuclear layer,* ONL* outer nuclear layer,* PL* photoreceptor layer

**Fig. 6:**
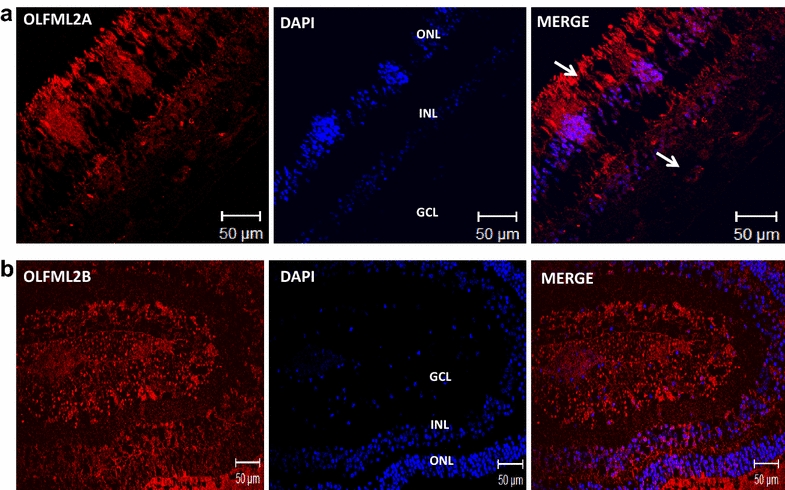
OLFML2A and OLFML2B immunoreactivity in human retina. **a** Confocal image of stained retina sections to identify cells expressing OLFML2A (*red*, 1st Ab: rabbit polyclonal anti-human OLFML2A 1:500; 2nd Ab: goat anti-rabbit IgG-Cy3® 1:4000). **b** Confocal image of stained retina seccions to identify cells expressing OLFML2B (*red*, 1st Ab: rabbit polyclonal anti-human OLFML2B 1:500; 2nd Ab: goat anti-rabbit IgG-Cy3® 1:4000). Cells nuclei were labeled with DAPI (*blue*). *GCL* ganglion cell layer,* INL* inner nuclear layer,* ONL* outer nuclear layer,* PL* photoreceptor layer

After identifying the ocular tissues which express both mRNAs, and in an effort to determine the cell type of the retina that expresses the genes, we performed the immunoreactivity (IR) analyses in baboon and human retina. IR signal of baboon retina for OLFML2A and OLFML2B are shown in Figs. 4 and 5 respectively. While in humans IR of OLFML2A and OLFML2B is observed in Fig. 6, also see Table 3. More analysis is required in normal human tissue. Only in baboon retina we did a double-immunolocalizaction of OLFML2A and OLFML2B with β-tubulin 3 beta chain, which is a cytoskeletal protein that is currently a neuronal cell marker in the developing and mature human nervous system to diferenciate retinal ganglion cells and astrocytes (Figs. 4, 5).

## Discussion

Olfactomedin was originally identified as the major component of the mucus layer that surrounds the chemosensory dendrites of olfactory neurons [15]. Subsequently, a vast numbers of proteins that share a ~250 amino acid domain homologous to olfactomedin were discovered in animals ranging from nematodes to humans [4]. One of these proteins was the olfactomedin like 2 proteins (OLFML2A and OLFML2B), also known as photomedins (−1 and −2, respectively), which were first identified and characterized in mouse retina [3]. But so far, it is not known if some primates such as baboon and human express these photomedins, whereas some olfactomedin-proteins like myocilin are associated with eye diseases such as glaucoma. Based on the above in the present study we cloned, sequenced and characterized the olfactomedin-like 2 cDNAs (OLFML2A and OLFML2B) in baboon (*Papio hamadryas*) and human from different ocular tissues. This is the first study that identifies expression of these genes in the eye of primates. Nevertheless, in mouse retina by northern blot analysis two RNA transcripts for OLFML2A (5 and 3.5 kb) and one for OLFML2B (3.5 kb) have been reported [15]. The authors suggest that these two RNA species for OLFML2A could be due to alternative splicing [3]. Also in human podocyte cells have been reported two OLFML2A mRNA variants [9]. However, we did not find in our study other transcripts of genes that may indicate the presence of isoforms derived by alternative splicing. This may be due to differences in the animal model studied; the tissues, and also the RNA transcripts found in mouse were not cloned and sequenced [3].

However, it is well known that some members of the subfamily of olfactomedin are expressed in the eye [3, 15, 16]. Similarly, the expression pattern suggests that mechanisms of regulation of gene expression are similar in the two species. It also suggests that the OLFML2A and OLFML2B genes might have similar physiological effects. However more studies are needed. The most extensively studied olfactomedin protein to date is myocilin (MYOC), which was first discovered in human trabecular meshwork cells [17, 18]. Several studies suggest that MYOC plays an important role in regulation in ocular hypertensin. Ocular hypertension is a major risk factor for glaucoma, leadin cause of blindness [15, 19]. Trabecular meshwork is a connective tissue that regulates the outflow at the iridocorneal angle of the eye and, hence, controls intraocular pressure [19], aqueous humor is continually produced by the ciliary body and it is in direct contact with the anterior surface of the lens, iris, and corneal endothelial cells, before draining out of the eye via the trabecular meshwork [20]. MYOC expression was observed in cornea, ciliary body, iris, sclera, optic nerve and retina, in human and mouse eye [21–23]. It is known that these tissues get their nutrientes from the aqueous humor and also export their metabolites, allowing an exchange with neighboring tissues [20]; it is also known that glaucoma is a group of progressive neurodegenerative multifactorial diseases, characterized by the loss of retinal ganglion cells (RGCs), optic nerve excavation, and axonal degeneration leading to irreversible vision loss [24]. Interestingly, we find the expression of photomedins (Table 3) in some of the tissues that express MYOC (cornea, lens, iris, and retina). The function of MYOC is still not known [19], however its been reported that MYOC may interact with other olfactomedin knows as optimedin (OLFM3), these wo proteins are expressed in human trabecular meswork and retina, and may be involved in glaucoma disease [25]. It would be interesting to study the interaction of myocilin and photomedins and see the correlation with these proteins in ocular pathologies.

The functional roles of the olfactomedin proteins in the retina are still not known [17]. Olfactomedins appear to be critical mediators for development of nervous systems and hematopoiesis [19]. Some others members are identified to be associated with human disease processes like glaucoma and cancer [4, 19]. Overexpression studies and inhibition of protein expression in zebrafish embryos showed that Noelin (olfactomedin 1), has profound effect on eye development, eye size, the projection field of retinal ganglion cells to the optic tectum, and extension and branching of retinal ganglion cell axons [19, 26]. Further studies showed in zebrafish that Noelin promotes retinal ganglion cell axon growth [27]. OLFM1 and OLFM2 are preferentially expressed in the developing retinal ganglion cells [16] in rat and mouse. In zebrafish eye OLFM2 was detected in the retinal ganglion cell layer and the inner nuclear layer [28]. OLFML2 in humans was found by RT-PCR in corneal endothelium, uvea, lens and retina-RPE. In baboon it was found in cornea, lens, iris, and retina-RPE [17]. Others authors have reported the expression of OLFM4 in mouse Müller glial cells. In the retina, OLFML2A was selectively expressed in the outer segment of photoreceptor cells and OLFML2B was expressed in all retinal neurons in a mouse. These proteins bound to other proteins like chondroitin sulphate-E and heparin suggest that photomedins-1 and -2 are extracellular proteins capable of binding of proteoglycans [3]. OLFML3 may play a possible role in angiogenesis in ocular tissues and it has been proposed that this protein may play a role in anterior segment and retinal diseases [17].

Positive selection (d_N_ > d_S_) implies that the substitutions, mostly non-synonymous, are functional and benefit the organism, conferring some evolutionary advantage. While purifying selection (d_N_ < d_S_) indicates that evolutionary pressure has been relaxed. The d_N_ and d_S_ rates show that the evolutionary force, actually acting on these, is the purification of the selection (P < 0.05). It fits the hypothesis that purifying of the selection is a clue that these genes are functional in the studied species, because there are not functional genes that do not fit this hypothesis. Similar expression profiles of human and baboon OLFML2A and OLFML2B genes, suggests that they have similar binding sites for known transcriptional factors. The phylogenetic relationship between NWM, OWM and apes OLFML2A proteins was determined to evaluate their evolution in primates. The phylogenetic tree (Fig. 1) shows three clades in a linage-specific manner. These clades correspond to NWM, OWM and apes, finally galago (out-group). The tree’s topology, branch length, and bootstrap values are similar using either phylogenetic method (ML/NJ/UPGMA). This confirms a clear orthology within the OLFML2A gene. While OLFML2B orthology is not clear (Fig. 2). The tree’s topology does not fit in a lineage-specific manner. It could be for many reasons, such as Ma’s night monkey sequence is shorter than the rest or it may be because more species should be included in the study.

Given these finding together, olfactomedins play essential roles in development and cell differentiation, also their effects are mediated through intercellular interactions, sometimes with other proteins or extracellular matrix components [15, 19], and some olfactomedin are implicated in important pathologies.

OLFML2A and OLFML2B seem to play an important role in ocular tissues, however the functions of these olfactomedins are still unknown. Therefore, further studies are needed to elucidate the role of these proteins in embryonic development, investigate its biological function, their protein interactions and diseases.

## Conclusions

The function of olfactomedin proteins in the eye, especially OLFML2A and OLFML2B, is still unknown; a lot of work is needed to clarify their actual role. Due to the high similarity between baboon and human olfactomedin expression, the baboon is a powerful model to deduce the physiological functions of these proteins in the eye.

## Abbreviations

OLF: olfactomedin
OLFML2A: olfactomedin like 2A
OLFML2B: olfactomedin like 2B
OLFML3: olfactomedin like 3
OLFML-1: olfactomedin like 1
aa: aminoacids
nt: nucleotides
bp: base pair
kb: kilo base
°C: celsius
RT-PCR: reverse transcription-polymerase chain reaction
cDNA: complementary deoxyribonucleic acid
RNA: ribonucleic acid
mRNA: messenger RNA
CDS: coding DNA sequence
min: minutes
sec: seconds
µl: microliter
mM: mili molar
nm: nanometer
IgG: inmmunoglobulin G
UV: ultraviolet
OWM: old world monkey
NWM: new world monkey
GCL: ganglion cell layer
IPL: inner plexiform layer
INL: inner nuclear layer
ONL: outer nuclear layer
RL: rod layer
PE: pigmented epithelium
